# Remarkable prevalence of coeliac disease in patients with irritable bowel syndrome plus fibromyalgia in comparison with those with isolated irritable bowel syndrome: a case-finding study

**DOI:** 10.1186/ar4391

**Published:** 2013-11-27

**Authors:** Luis Rodrigo, Ignacio Blanco, Julio Bobes, Frederick J de Serres

**Affiliations:** 1 Gastroenterology, Central University Hospital of Asturias (HUCA), Celestino Villamil, s/n. ES-33006 Oviedo, Principality of Asturias, Spain; 2 Biomedical Research Office of the Principality of Asturias, FICYT, c/Rosal 7–bis, ES-33009 Oviedo, Principality of Asturias, Spain; 3 Medicine Department, Psychiatry Area, University of Oviedo, Julian Clavería 6, ES-33006 Oviedo, Principality of Asturias, Spain; 4 National Institute of Environmental Health Sciences, Research Triangle Park, NC 27709-2233, USA

## Abstract

**Introduction:**

Irritable bowel syndrome (IBS) and fibromyalgia syndrome (FMS) are two common central sensitization disorders frequently associated in the same patient, and some of these patients with IBS plus FMS (IBS/FMS) could actually be undiagnosed of coeliac disease (CD). The present study was an active case finding for CD in two IBS cohorts, one constituted by IBS/FMS subjects and the other by people with isolated IBS.

**Methods:**

A total of 104 patients (89.4% females) fulfilling the 1990 ACR criteria for FMS and the Rome III criteria for IBS classification and 125 unrelated age- and sex-matched IBS patients without FMS underwent the following studies: haematological, coagulation and biochemistry tests, serological and genetic markers for CD (i.e., tissue transglutaminase 2 (tTG-2) and major histocompatibility complex HLA-DQ2/HLA-DQ8), multiple gastric and duodenal biopsies, FMS tender points (TPs), Fibromyalgia Impact Questionnaire (FIQ), Health Assessment Questionnaire (HAQ), 36-Item Short Form Health Survey (SF-36) and Visual Analogue Scales (VASs) for tiredness and gastrointestinal complaints.

**Results:**

As a whole, IBS/FMS patients scored much worse in quality of life and VAS scores than those with isolated IBS (*P* < 0.001). Seven subjects (6.7%) from the IBS/FMS group displayed HLA-DQ2/HLA-DQ8 positivity, high tTG-2 serum levels and duodenal villous atrophy, concordant with CD. Interestingly enough, these seven patients were started on a gluten-free diet (GFD), showing a remarkable improvement in their digestive and systemic symptoms on follow-up.

**Conclusions:**

The findings of this screening indicate that a non-negligible percentage of IBS/FMS patients are CD patients, whose symptoms can improve and in whom long-term CD-related complications might possibly be prevented with a strict lifelong GFD.

## Introduction

Irritable bowel syndrome (IBS) is a common gastrointestinal functional disease characterized by the presence of chronic abdominal pain or discomfort associated with changes in bowel habits, consisting predominantly of diarrhoea, constipation or alternating patterns; defecation urgency; tenesmus; bloating; and abdominal distension [1,2]. Diagnosis of IBS is based on a positive history of gastrointestinal symptoms according to the Rome III criteria in the absence of obvious alarm signs [3].

Several comorbidities which may occur more often than expected by chance in IBS patients have been identified, including fibromyalgia syndrome (FMS), chronic fatigue syndrome (CFS), gastro-oesophageal reflux disease, headache, backache, genitourinary symptoms, temporomandibular joint disorder, anxiety and depression [4,5]. Specifically, FMS, a prevalent, chronic, widespread pain disorder affecting mainly females, classified according to the 1990 American College of Rheumatology (ACR) criteria [6], occurs in 20% to 32% of people with IBS, and, in turn, 32% to 70% of people with FMS also meet the criteria for IBS [7-9]. It has been reported that the incidence of coeliac disease (CD) in patients with IBS is much higher than that expected to be found in the general population [10,11].

In spite of the fact that IBS, FMS and CD are three prevalent disorders in Spain, no study has been carried out to date to assess their association rate in this country. Therefore, with the aim of estimating the prevalence of CD masquerading as IBS and/or FMS, a specific protocol for active case-finding of CD was applied to an IBS/FMS population from Asturias (Cantabrian coast, northern Spain) and to a sex-, age- and size-matched cohort of IBS patients without FMS to establish comparisons.

## Methods

During the 6-year period from 2007 through 2012, the Rome III criteria for IBS diagnosis [3] and the 1990 ACR criteria for FMS classification [6] were prospectively applied to 442 consecutive patients upon their first visit to an outpatient gastroenterology clinic at the Central University Hospital of Asturias (HUCA; Oviedo, Spain). Most patients were referred from the rheumatology and internal medicine departments of the same hospital for the study of a variety of long-standing gastrointestinal symptoms.

Diagnosis of IBS was based on a positive history of abdominal discomfort or pain associated with disturbed defecation (according to the Rome III criteria) in the absence of obvious alarm features such as rectal bleeding, iron deficiency anaemia, weight loss, fever, onset after age 40 years, family history of colon cancer, nocturnal symptoms and faecal soilage. When these symptoms are present, a more extensive evaluation must be carried out in the differential diagnosis. The evaluation should include testing for coeliac sprue, structural colon lesions such as polyps or cancer, parasites, endocrine disease, bacterial overgrowth or carbohydrate malabsorption.

Despite the fact that IBS is no longer a diagnosis of exclusion, since there is good evidence that a positive clinical diagnosis is reliable enough [12], a battery of tests was performed in all patients before they were entered into the study, including a comprehensive medical history, a thorough physical examination and complete laboratory haematological and biochemical broad screening.

In patients who did not respond to the usual therapies, as well as in IBS cases in which other associated organic illnesses, a specific hydogren breath test was performed in order to exclude possible lactose intolerance or small-bowel bacterial overgrowth. Appropriate faecal cultures were done in some patients to exclude the presence of parasitic infections. Furthermore, in patients with persistent diarrhoea, a total colonoscopy was performed and random colonic biopsies were taken to rule out microscopic colitis.

An immunological faecal occult blood test (iFOBT) was done in patients over 50 years of age and in those with a positive familial history of colon cancer in first-degree relatives. If this iFOBT was positive, the study was completed with a total colonoscopy.

The acceptance criteria for IBS patients included (1) older than 18 and younger than 65 years of age, (2) meeting the Rome III criteria, (3) absence of any other associated organic gastrointestinal disease and (4) not meeting the 1990 ACR criteria for FMS. These selection criteria were also used to select IBS patients with associated FMS, except the last one, because by definition they had to fulfil the 1990 ACR criteria for FMS classification. The exclusion criteria were (1) incomplete or doubtful Rome III criteria for IBS cases and incomplete or doubtful 1990 ACR criteria for IBS/FMS cases, (2) any abnormal finding in the analytical screening or in the colonoscopy or colonic biopsies and (3) unwillingness to participate in the study.

Only 263 (59%) of 442 individuals were eligible to participate in the study, and 34 of the eligible people did not agree to sign a written consent form, thus excluding them from participation. Thus a total of 229 individuals agreed to take part in this study. Participants were then assigned to two groups: 104 comprising the IBS plus FMS group, who fulfilled both the Rome III criteria for IBS diagnosis and the 1990 ACR criteria for FMS classification, and 125 constituted the IBS group, who were unrelated age- and sex-matched patients from the same Asturias population who met the Rome III criteria for IBS diagnosis but did not have FMS widespread pain or skin tender points (TPs) and did not meet the 1990 ACR criteria.

These 229 participants were invited to participate on a voluntary basis after signing a specific informed consent form. The study was approved by the HUCA Research and Ethics Committee according to the principles included in the modified Declaration of Helsinki.

### Outline of the study protocol

Initially, every selected participant underwent an updated medical history, a quality of life (QoL) battery of tests and a thorough physical examination.

#### ***Tender points***

The FMS TPs were identified by digit pressure on the 18 locations recommended by the 1990 ACR criteria [6]. This test was routinely carried out in the clinical setting. Digital palpation with the thumb pad was performed on the standard TP sites on a copy of the body figure (front and back) with location of all TPs that we found in each patient included in the clinical record. An approximate force of 4 kg, sufficient to blanch the nail bed of the thumb, was applied over each of these TPs. The TPs were palpated with constant palpation for about 4 seconds. Patients responded ‘yes’ or ‘no’ if they had any pain. When they responded ‘yes’ , the examiner asked them to rate their pain on a scale of 0 (no pain) to 10 (worst pain) and recorded each response on the body figure.

### Physical, mental, psychological, social functioning and quality of life questionnaires

Each participant filled out the self-administered Spanish version form of the Fibromyalgia Impact Questionnaire (FIQ) [13], the Stanford Health Assessment Questionnaire (HAQ) [14] and the 36-Item Short Form Health Survey (SF-36) [15].

The FIQ is a 10-item instrument that measures, in a range from 0 to 80 points, the physical functioning, work status and degree of depression, anxiety, sleep, pain, stiffness, fatigue and well-being, with scores of 0 to 39, 40 to 59 and 60 or greater evaluated as mild, moderate and severe FMS, respectively.

The 20-item disability scale portion of the HAQ measures, in a range from 0 to 3 points, the patient’s difficulty with activities of daily living and concomitant need for help and assistive devices, with the highest scores representing the maximum impairment (that is, 0 = able to do without any difficulty, 1 = some difficulty, 2 = much difficulty and 3 = unable to do).

The SF-36 is has two components: the Physical Component Summary (PCS) and the Mental Component Summary (MCS). Scores in the range from 0 to 100 punctuate each of these aspects, with the lowest scores indicating the poorest health status. No cutoff values on the SF-36 for classifying the impact of disease have been established yet. For guidance, the reported values for adults in the Spanish general population (expressed as mean and standard deviation) [16] were PCS 73.0 (27.8) and MCS 74.4 (24.4).

#### ***Laboratory tests***

Complete blood cell counts were performed with the CELL-DYN 3500 automated haematology analyser (Abbott Cientifica, Madrid, Spain). Coagulation panels were carried out with the IL ACL 3000 Coagulation Analyzer (Beckman Coulter, Brea, CA, USA). Analytical biochemistry tests were performed on a Roche Hitachi automated modular chemistry analyser (Roche Diagnostic Systems, Indianapolis, IN, USA) for SXA-phosphoprotein-binding domains using enzymatic or kinetic methods, including urea, glucose, total proteins, albumin, C-reactive protein, calcium, folate, vitamin B_12_, creatinine, creatine kinase, lipid profile, liver function tests (LFTs), iron metabolism tests, immunoglobulin G (IgG), IgA and IgM, rheumatoid factor, thyroid function test and urinalysis with microscopic examination of sediment.

Antinuclear antibodies (ANAs) and antithyroid peroxidase (anti-TPO) antibodies were measured in each participant, and in those individuals with altered LFTs antimitochondrial antibodies (AMAs) were also assessed. Determination of these biomarkers was performed by indirect immunofluorescence assay on the HEp-20-10 cell line according to the manufacturer’s instructions (EUROIMMUN, Lübeck, Germany). Anti-IgA tissue transglutaminase subtype 2 (tTG-2) was measured by using a commercially available enzyme-linked immunosorbent assay kit (Phadia Diagnostics, Uppsala, Sweden).

To assess the participants’ genetic susceptibility to CD, the major histocompatibility complex class II human leucocyte antigen (HLA) marker HLA-DQ2 was characterized by means of a polymerase chain reaction (PCR) with a commercially available kit (PROTRANS HLA Celiac Disease Domino System; PROTRANS, Hockenheim, Germany). Characterization of the HLA-DQ8 haplotype was not systematically assayed in this explorative study, but it was carried out in isolated HLA-DQ2-negative cases showing villous atrophy in duodenal biopsy.

#### ***Duodenal biopsy studies***

An upper gastrointestinal endoscopy with at least four duodenal biopsies was performed in all patients following the usual methodology employed in our service for CD diagnosis [17]. Samples were routinely stained with haematoxylin and eosin (H & E) and with anti-CD3 immunohistochemical monoclonal antibodies to count the number of intraepithelial lymphocytes (IELs) and in turn to quantify them per 100 epithelial cells. Samples were studied by two expert pathologists at HUCA and classified into the following types according to the histological classification for CD described by Marsh [18] and later modified by Oberhüber *et al*. [19]: stage 0: histological normal duodenum; stage 1: increased IEL infiltration with a total count of 25% or greater; stage 2: crypt hyperplasia and diffuse chronic inflammatory infiltrate at the lamina propria; and stage 3: villous atrophy, which was subdivided into three categories: (a) mild, (b) moderate and (c) severe.

*Helicobacter pylori* was systematically investigated by taking endoscopic biopsies from the antrum (*n* = 2) and the corpus of the stomach (*n* = 2). Antibiotics, proton pump inhibitors (PPIs) and drugs containing bismuth and H2 blockers were stopped within the previous 2 weeks. One antral biopsy was immediately used for a quick urease test (Pronto Dry Kit; Pentland Medical Ltd, Edinburgh, UK). The rest of the samples were used for histopathological examination (that is, routine H & E staining, Giemsa staining and immunohistochemistry using polyclonal anti-*H. pylori* antibody) and microbial cultures. Positive cases received triple therapy comprising 14-day treatment with PPI (standard dose twice daily), clarithromycin (500 mg twice daily) and amoxicillin (1,000 mg twice daily). After treatment was completed, *H. pylori* eradication was confirmed 4 to 6 weeks later with a rapid urease breath test [20,21].

### Statistical analysis

Descriptive statistics (means calculations, standard deviations and observed ranges) were used on continuous parameters. For qualitative variables, percentages were used. Kruskal–Wallis analysis of variance (ANOVA) contingency tables were analysed. If the continuous variables followed a normal distribution, Student’s *t*-test was used. Differences between groups were evaluated by ANOVA followed by *post hoc* analysis using Fisher’s test. The statistical calculations were performed using SPSS 15.0 software (SPSS Inc, Chicago, IL, USA), and *P* values less than 0.05 were considered significant.

## Results

### Demographics

The 229 participants in the study were unrelated Caucasians from the Principality of Asturias, Spain. No statistical differences among groups were found regarding age, gender, marital status, labour status or educational level. Participants included in the IBS/FMS Marsh stage 1 group had a slightly higher body mass index indicative of moderate overweight. Ten patients from the FMS/IBS group classified as Marsh stage 1 and two classified as Marsh stage 3 had a total of 12 first-degree relatives (that is, 3 parents, 6 siblings and 3 offspring) previously diagnosed with CD. Five patients from the FMS/IBS group and four from the IBS group declared that they had five and four siblings, respectively, diagnosed by other physicians as FMS patients (Table 1).

**Table 1:** **Demographic data**^**a**^

				**IBS/FMS cases**			
			***P *** **value**	**Classified by Marsh stage**			***P *** **value**
	**IBS**	**IBS/FMS**	**(IBS vs. FMS)**	**Marsh stage 0**	**Marsh stage 1**	**Marsh stage 3**	**(Marsh stages 1 and 3 vs. Marsh stage 0)**
Sample size	125	104	NA	39	58	7	<0.001
Females	104 (84)	93 (89)	0.122	34 (87)	52 (90)	7 (100)	0.595
Age, years	51 (8)	50 (8)	0.668	49 (7)	51 (9)	49 (12)	0.146
BMI, kg/m^2^	27 (4)	26 (4)	0.388	25 (3)	28 (5)	24 (3)	0.003
Marital status							
Single	3 (2)	2 (2)	0.748	1 (3)	2 (3)	1 (14)	0.892
Married	103 (82)	92 (89)	0.272	33 (85)	52 (90)	4 (57)	1.000
Domestic partnership	10 (8)	5 (5)	0.481	4 (10)	1 (0)	1 (14)	0.123
Separated/divorced	10 (8)	5 (5)	0.481	1 (3)	3 (5)	1 (14)	0.722
Education level							
None	2 (2)	2 (2)	0.748	2 (5)	2 (3)	1 (14)	0.834
Primary school	92 (73)	75 (72)	0.918	29 (74)	42 (72)	2 (28)	0.865
Secondary education	27 (22)	24 (23)	0.914	10 (26)	11 (19)	3 (42)	0.810
Higher education	4 (3)	3 (3)	0.804	3 (8)	3 (5)	1 (14)	0.449
Labour status							
Employed	59 (47)	47 (45)	0.864	21 (54)	24 (41)	2 (29)	0.242
Unemployed	26 (21)	22 (21)	0.922	7 (18)	13 (22)	2 (29)	0.844
Householder chores	23 (18)	19 (18)	0.883	8 (20)	9 (15)	2 (29)	0.709
Retired/pensioner	17 (14)	16 (15)	0.846	3 (8)	12 (21)	1 (14)	0.160
Participants with relatives diagnosed with CD	–	12 (11)	NA	2 (5)	10 (17)	2 (29)	0.161
Participants with relatives diagnosed with FMS	4 (3)	5 (5)	0.389	1 (2)	4 (7)	1 (14)	0.619

^a^BMI, body mass index; CD, coeliac disease; FMS, fibromyalgia syndrome; IBS, irritable bowel syndrome; NA, not applicable. Data are expressed as total and percentage of the total value or mean and standard deviation.

### Duodenal biopsy histological findings and serological and genetic markers of coeliac disease

IEL counts were significantly higher in the IBS/FMS group than in the IBS group (odds ratio (OR) = 2.577; 95% confidence interval (CI) = 1.283 to 5.154). The prevalence of Marsh stage 1 cases was higher in the IBS/FMS cohort, and seven cases showed villous atrophy (classified as Marsh stage 3) compared with none in the IBS group, with clear differences observed between both groups (OR = 8.750; 95% CI = 4.699 to 16.289).

About half of the patients in both groups were infected with *H. pylori* (42% in the IBS/FMS group and 46% in the isolated IBS group) and treated with 14-day triple therapy. *H. pylori* eradication was confirmed in 90% of IBS/FM cases and in 88% of isolated IBS cases by the urease breath test, without any differences in prevalence and eradication percentages observed between them. Fifty percent of FMS/IBS patients vs. twenty-four percent from the IBS group were HLA-DQ2-positive, with a rising gradient of positivity proportional to the degree of histological damage, that is, Marsh stage 0 (38%) < Marsh stage 1 (53%) < Marsh stage 3 (86%). A HLA-DQ2-negative case with lymphocytic infiltration and villous atrophy in the duodenal mucosa was found to be HLA-DQ8-positive. High values of IgA anti tTG-2 titres were found only in the seven IBS/FMS cases classified as Marsh stage 3 (Tables 2 and 3).

**Table 2:** **Gastroduodenal histological findings and genetic and serological markers**^**a**^

**Cohorts**	**IBS (*N*** **= 125)**	**IBS/FMS (*N*** **= 104)**	***P *** **value**
Gastroduodenal biopsy			
IEL count per 100 epithelial cells	15 (10)	27 (26)	<0.001
Marsh stage 0	105 (84)	39 (37)	<0.001
Marsh stage 1	20 (16)	58 (56)	<0.001
Marsh stage 3	2 (2)	7 (7)	<0.001
*Helicobacter pylori* (+)	58 (46)	44 (42)	0.080
CD laboratory biomarkers			
HLA-DQ2 A1/B1 (+)	30 (24)	52 (50)	<0.001
IgA anti-tTG-2 serum levels, U/ml	0.4 (0.1)	4.7 (20)	<0.001

^a^CD, coeliac disease; HLA, major histocompatibility complex class II human leucocyte antigen; IBS, irritable bowel syndrome; IBS/FMS, irritable bowel syndrome plus fibromyalgia syndrome; IEL, intraepithelial lymphocyte count per 100 epithelial cells in duodenal biopsy sample; IgA, immunoglobulin A; tTG, tissue transglutaminase. Data are expressed as total and percentage of the total value or as mean and standard deviation.

**Table 3:** **Laboratory results in inflammatory bowel syndrome and fibromyalgia syndrome subgroups**^**a**^

**Laboratory test**	**IBS**	**IBS/FMS cases classified by Marsh stage**			**P value**
	**(*N*** **= 125)**	**(*N*** **= 104)**			**(Marsh stages 1 and 3 vs. Marsh stage 0)**
		**Normal mucosa**	**Lymphocytic enteritis**	**Villous atrophy**	
		**Marsh stage 0 (*n*** **= 39)**	**Marsh stage 1 (*n*** **= 58)**	**Marsh stage 3 (*n*** **= 7)**	
IEL count per 100 epithelial cells	15 (10)	14 (2)	35 (5)	37 (5)	<0.001
*Helicobacter pylori* (+)	58 (46)	17 (43)	24 (41)	3 (43)	0.159
HLA-DQ2 A1/B1 (+)	30 (24)	15 (38)	31 (53)	6 (86)	0.052
IgA anti tTG-2*,* U/ml	0.4 (0.1)	0.5 (0.1)	0.9 (0.7)	60.4 (52)	0.001

^a^HLA, major histocompatibility complex class II human leucocyte antigen; IBS, irritable bowel syndrome; IBS/FMS, irritable bowel syndrome plus fibromyalgia syndrome; IEL, intraepithelial lymphocyte count per 100 epithelial cells in duodenal biopsy sample; IgA, immunoglobulin A; tTG, tissue transglutaminase. Data are expressed as mean and standard deviation or total and percentage of the total value.

### Gastrointestinal symptoms

All patients complained of a combination of at least two of the following gastrointestinal symptoms: abdominal pain or discomfort, bloating, heartburn, constipation, diarrhoea and alternating diarrhoea and constipation. The duration of symptoms was long-lasting in both groups, without statistical differences between them (Table 4).

**Table 4:** **Main symptoms, associated diseases, tender points and test scores**^**a**^

**Symptoms and diseases**	**IBS**	**IBS/FMS**	***P *** **value**	**IBS/FMS cases classified by Marsh stage**			***P *** **value**
	**(*N*** **= 125)**	**(*N*** **= 104)**	**(IBS vs. FMS)**	**Marsh stage 0 (*N*** **= 39)**	**Marsh stage 1 (*N*** **= 58)**	**Marsh stage 3 (*N*** **= 7)**	**(Marsh stages 1 and 3 vs. Marsh stage 0)**
Widespread pain	–	104 (100)	NA	39 (100)	58 (100)	7 (100)	1.000
Digestive complaints	125 (100)	104 (100)	1.000	39 (100)	58 (100)	7 (100)	1.000
Fatigue	31 (25)	94 (90)	<0.001	35 (90)	53 (91)	6 (86)	0.878
Sleep disturbances	28 (22)	87 (84)	<0.001	29 (74)	52 (89)	6 (86)	0.134
Anxiety/depression	77 (62)	69 (66)	0.273	22 (56)	42 (72)	5 (71)	0.251
Skin problems^b^	31 (25)	52 (50)	<0.001	20 (51)	28 (48)	4 (57)	0.888
Cognitive dysfunction	5 (4)	37 (36)	<0.001	14 (36)	20 (34)	3 (43)	0.908
Urinary urgency	6 (5)	30 (29)	<0.001	10 (26)	18 (31)	2 (29)	0.848
Headaches^c^	24 (19)	29 (28)	0.082	5 (13)	21 (36)	3 (43)	0.028
Balance problems/dizziness	9 (7)	29 (28)	<0.001	10 (26)	17 (29)	2 (29)	0.924
Joint stiffness	2 (2)	27 (26)	<0.001	9 (23)	16 (28)	2 (29)	0.872
Paraesthesias	4 (3)	25 (24)	<0.001	9 (23)	14 (24)	2 (29)	0.952
Osteoporosis	7 (6)	14 (13)	0.034	2 (5)	10 (17)	2 (29)	0.110
TMJ disorder	–	13 (12)	NA	5 (13)	6 (10)	2 (29)	0.520
Restless legs syndrome	–	13 (12)	NA	3 (8)	8 (14)	1 (14)	0.277
Sjögren syndrome	–	8 (8)	NA	–	7 (12)	1 (14)	NA
Raynaud syndrome	1 (1)	5 (5)	0.069	–	5 (9)	1 (14)	NA
Tender points (scale 0 to 18)	–	16.1 (1.9)	NA	14.8 (1.2)	16.9 (1.8)	16.3 (2.4)	<0.001
FIQ score (scale 0 to 80)	–	68 (9)	NA	63 (7)	70 (9)	74 (3)	<0.001
HAQ score (scale 0 to 3)	–	1.5 (0.6)	NA	1.4 (0.5)	1.6 (0.6)	1.7 (0.6)	0.298
SF-36 Physical Component (scale 0 to 100)	44 (13)	29 (5)	<0.001	30 (7)	29 (1)	27 (4)	0.380
SF-36 Mental Component (scale 0 to 100)	41 (13)	29 (14)	<0.001	35 (10)	25 (13)	17 (4)	<0.001
VAS fatigue score (scale 0 to 10)	3 (1)	7 (2)	<0.001	7 (0.8)	8 (1.5)	8 (0.3)	0.006
VAS digestive score (scale 0 to 100)	31 (12)	47 (8)	<0.001	46 (9)	47 (9)	48 (2)	0.516
Gastrointestinal symptoms duration, years	29 (5)	29 (7)	0.917	29 (5)	29 (8)	28 (9)	0.905
Extraintestinal systemic symptom duration, years	–	9 (2)	NA	9 (2)	9 (2)	7 (4)	0.813

^a^FIQ, Fibromyalgia Impact Questionnaire; HAQ, Health Assessment Questionnaire; IBS, inflammatory bowel syndrome; IBS/FMS, inflammatory bowel syndrome/fibromyalgia syndrome; NA, not applicable; SF-36, 36-Item Short Form Health Survey; TMJ = temporomandibular joint; VAS, Visual Analogue Scale. ^b^Skin problems included itchy, dry or burning skin; chronic urticaria; and one case of dermatitis herpetiformis that was classified as Marsh stage 3. ^c^Headaches included migraine, tension and mixed headache syndrome. Data are expressed as total and percentage of total values or mean and standard deviation.

### Fibromyalgia symptoms

IBS/FMS patients had a number of classical FMS symptoms, starting in most patients during their mid-40s. Except for headaches, which were more frequent in the Marsh stages 1 and 3 subgroups, the symptoms appeared to be homogeneously distributed among the Marsh stage subgroups (Table 4).

### Tender points and quality of life questionnaires

IBS/FMS patients scored very high in TPs and on the FIQ and HAQ scales and, conversely, very low on the SF-36 items. As a whole, these patients were classified as having severe FMS. Patients with only IBS displayed significantly better scores than the IBS/FMS patients on the SF-36 and the VAS digestive and fatigue scales (Table 4).

### Pharmacological therapy

Practically all patients received prescriptions for antispasmodic, antidiarrhoeal or laxative medications. The use of anxiolytic and antidepressant medications was substantial in both groups, reflecting the high prevalence of anxiety and depression in these patients. The use of analgesics, antidepressants, benzodiazepines, hypnotics, pregabalin, laxatives and opiate patches, as well as the number of drugs prescribed per patient per day, were significantly higher in the IBS/FMS group than in patients with only IBS, specifically in the Marsh stages 1 and 3 subgroups (Table 5).

**Table 5:** **Most frequently prescribed drugs**^**a**^

**Prescribed drugs**	**IBS**	**IBS/FMS**	***P *** **value**	**IBS/FMS cases classified by Marsh stage**			***P *** **value**
	**(*N*** **= 125)**	**(*N*** **= 104)**	**(BIS vs. FMS)**	**Marsh stage 0 (*N*** **= 39)**	**Marsh stage 1 (*N*** **= 58)**	**Marsh stage 3 (*N*** **= 7)**	**(Marsh stages 1 and 3 vs. Marsh stage 0)**
Analgesics^b^	22 (18)	85 (82)	<0.001	34 (87)	45 (78)	6 (86)	0.468
Omeprazole	102 (82)	82 (79)	0.360	29 (74)	47 (81)	6 (86)	0.659
Antidepressants^c^	30 (24)	70 (67)	<0.001	25 (64)	40 (69)	5 (71)	0.857
Pregabalin	4 (3)	60 (58)	<0.001	19 (49)	36 (62)	5 (71)	0.319
Benzodiazepines/hypnotics	55 (44)	59 (57)	0.037	20 (51)	35 (60)	4 (57)	0.677
Antispasmodics/antidiarrhoeals^d^	58 (46)	52 (50)	0.341	17 (38)	33 (57)	2 (29)	0.220
Laxatives	14 (11)	42 (40)	<0.001	10 (26)	27 (47)	5 (71)	0.027
Opiate patches	–	17 (16)	NA	1 (2)	12 (21)	4 (57)	<0.001
Number of drugs prescribed per patient per day	1.8 (1.1)	4.6 (2.2)	<0.001	3.9 (1.9)	4.9 (2.2)	6.6 (1.5)	0.003

^a^IBS, inflammatory bowel syndrome; IBS/FMS, inflammatory bowel syndrome/fibromyalgia syndrome; NA = not applicable. ^b^Analgesics were usually used on an irregular basis (on demand) and were generally nonsteroidal anti-inflammatory drugs, acetaminophen, tramadol, metamizole and codeine. ^c^Antidepressants prescribed were tricyclic antidepressants, selective serotonin reuptake inhibitors or other antidepressants. ^d^Antispasmodics prescribed were mebeverine and/or octylonium bromide, and antidiarrhoeal medications prescribed were loperamide and diphenoxylate/atropine. Data are expressed as total value and percentage of the total value or mean and standard deviation.

### Haematological, biochemical and immunological laboratory tests

Overall, mean values for the different general haematological and biochemical analyses were normal or slightly altered, with a wide dispersion and broad range (Table 6).

**Table 6:** **Haematological and biochemical laboratory findings**^**a**^

**Laboratory panels**	**IBS**	**IBS/FMS**	***P *** **value**	**IBS/FMS cases classified by Marsh stage**			***P *** **value**
	**(*N*** **= 125)**	**(*N*** **= 104)**	**(IBS vs. FMS)**	**Marsh stage 0 (*N*** **= 39)**	**Marsh stage 1 (*N*** **= 58)**	**Marsh stage 3 (*N*** **= 7)**	**(Marsh stages 1 and 3 vs. Marsh stage 0)**
Haemoglobin, g/dl	13 ( 1)	13 (1)	1.000	13 (1)	14 (1)	13 (1)	0.271
WBC count, *n* × 10^3^	7.6 (1)	7.0 (2)	0.002	7.6 (1)	6.7 (2)	5.8 (2)	0.002
Platelet count, *n* × 10^3^	272 (37)	257 (54)	0.021	274 (37)	248 (60)	232 (57)	0.010
Prothrombin activity (%)	98 (5)	98 (9)	0.777	98.2 (8)	98 (10)	98 (4)	0.741
aPTT, seconds	34 (2)	35 (2)	0.024	35 (2)	33 (2)	33 (1)	0.019
Fibrinogen, mg/dl	261 (111)	349 (152)	<0.001	259 (113)	385 (128)	552 (218)	<0.001
Glucose, mg/dl	108 (27)	101 (20)	0.018	109 (27)	96 (13)	99 (9)	0.020
Urea, mg/dl	44 (23)	40 (14)	0.022	43 (21)	38 (7)	38 (8)	0.017
CK, IU/L	59 (7)	60 (9)	0.985	58 (7)	61 (10)	62 (12)	0.276
CRP, mg/L	0.6 (0.6)	0.5 (0.6)	0.211	0.6 (0.7)	0.5 (0.7)	0.3 (0.2)	0.466
IgG, g/L	9.7 (0.5)	9.9 (1.0)	0.098	9.6 (0.5)	10.2 (1.2)	9.6 (1.0)	0.107
IgA, g/L	2.4 (0.5)	2.6 (0.6)	0.126	2.4 (0.6)	2.7 (0.6)	2.4 (0.5)	0.008
IgM, g/L	1.8 (0.4)	1.9 (0.5 )	0.126	1.9 (0.3)	1.9 (0.6)	1.6 (0.5)	0.722
Iron, μg/ml	68 (19)	80 (29)	0.001	66 (20)	89 (32)	91 (21)	<0.001
Ferritin, ng/ml	56 (23)	88 (164)	0.821	48 (28)	114 (214)	90 (70)	0.064
TSI, μg/dl	20 (11)	24 (9)	0.001	20 (11)	25 (8)	25 (6)	0.001
Cholesterol, g/dl	176 (24)	174 (39)	0.293	192 (18)	166 (42)	144 (52)	0.001
AST, IU/L	20 (9)	23 (9)	<0.001	21 (5)	25 (11)	30 (20)	0.269
ALT, IU/L	21 (6)	25 (13)	0.803	21 (7)	27 (17)	19 (10)	0.765
GGT, IU/L	23 (20)	28 (23)	0.074	24 (20)	31 (22)	23 (33)	0.060
ALP, IU/L	70 (23)	76 (26)	0.012	74 (30)	78 (23)	77 (22)	0.066
Bilirubin, mg/dl	0.7 (0.2)	0.8 (0.2)	0.639	0.7 (0.1)	0.7 (0.2)	0.9 (0.24)	0.591
TSH, μU/ml	1.8 (0.6)	3.1 (5.9)	0.209	1.9 (0.7)	4.1 (7.8)	1.2 (0.8)	0.157
Folate, ng/dl	10 (4)	9 (3)	0.019	10 (3)	8 (3)	10 (5)	0.019
Vitamin B_12_, pg/ml	451 (168)	521 (206)	0.002	450 (170)	555 (213)	636 (238)	0.001
RF, IU/ml	13 (1)	13 (2)	0.858	13 (1)	13 (2)	14 (2)	0.775
ANAs (+)	4 (3)	29 (28)	<0.001	3 (8)	23 (40)	3 (43)	0.002
Titres							
1:160	4 (3)	23 (22)	<0.001	4 (10)	18 (31)	1 (14)	0.004
1:320	–	3 (3)	NA	–	2 (3)	1 (14)	NA
1:640	–	3 (3)	NA	–	2 (3)	1 (14)	NA
Staining pattern							
Speckled	4 (3)	25 (24)	<0.001	4 (10)	21 (34)	–	NA
Homogeneous	–	2 (2)	NA	–	1 (2)	1 (14)	NA
Nucleolar	–	2 (2)	NA	–	–	2 (29)	NA
Anti-TPO (+)	–	12 (11)	NA	–	10 (17)	2 (29)	0.011
Serum levels, IU/ml	–	31 (108)	NA	–	51 (140)	40 (72)	NA
AMAs (+)	–	5 (4)	NA	–	4 (7)	1 (0)	NA
Titres							
1:160	–	1 (1)	NA	–	1 (2)	–	NA
1:320	–	2 (2)	NA	–	1 (3)	1 (14)	NA
1:640	–	2 (2)	NA	–	2 (3)	–	NA

^a^ALP, alkaline phosphatase; ALT, alanine aminotransferase; AMA, antimitochondrial antibody; ANA, antinuclear antibody; Anti-TPO, antithyroid peroxidase antibody; aPTT, activated partial thromboplastin time; AST, aspartate aminotransferase; CK, creatine kinase; CRP, C-reactive protein; GGT, γ-glutamyl transpeptidase; Ig, immunoglobulin; NA, not applicable; RF, rheumatoid factor; TSH, thyroid-stimulating hormone; TSI, Transferrin Saturation Index; WBC, white blood cell. Values are expressed as mean and standard deviation or total value and percentage.

ANAs were positive, with variable titres and patterns, in 29 IBS/FMS cases, most of which were classified as Marsh stages 1 and 3, compared with 4 ANA-positive patients with IBS alone (*P* < 0.001). However, none of the ANA-positive patients met the 1982 ACR criteria necessary for the diagnosis of systemic lupus erythematous (SLE) [22].

High serum levels of anti-TPO antibodies were found in ten Marsh stage 1 cases and two Marsh stage 3 cases in the IBS/FMS group. Nevertheless, only five of these positive cases showed minor, transitory thyroid function test alterations, compatible with hypothyroidism in four cases and with hyperthyroidism in one case, but none of these showed clinical manifestations of thyroid dysfunction.

AMAs were positive in five IBS/FMS cases (four were Marsh stage 1 and one was Marsh stage 3). Each of these patients presented with mild elevation of serum aspartate aminotransferase, alanine aminotransferase, alkaline phosphatase and γ-glutamyl transpeptidase levels, but without clinical manifestations of liver disease, and thus were diagnosed as having subclinical autoimmune hepatitis.

## Discussion

The most remarkable finding of this pilot study was our finding of seven cases with solid histological, laboratory and clinical evidence of CD in a group of FMS patients (mostly females) whose clinical symptoms started with gastrointestinal relapsing disorders mimicking IBS when they were in their 20s. They developed multisystem complaints about two decades later, with very negative effects in their health-related QoL. Remarkably, almost 30% of these CD-associated IBS and FMS patients had a family history of CD, and a significant number of them also had high serum titres of ANAs, anti-TPOs and AMAs. Interestingly enough, these seven patients were started on a gluten-free diet (GFD), which led to significant improvement in their digestive and systemic symptoms upon follow-up examinations.

A recent systematic review concluded that biopsy-proved CD in cases meeting the diagnostic criteria for IBS was more than fourfold higher than that in controls without IBS [23]. Although further work is needed to definitively confirm that CD is a significant problem in terms of misdiagnosis in IBS, it has been estimated that testing IBS patients for CD can be cost-effective in clinical practice [24].

In recent years, a possible causal link between IBS, FMS and multisymptom forms of adult CD has been suspected on the basis of some cases with overlapping symptoms involving gastrointestinal, musculoskeletal and other body systems that have had similar clinical features [25,26]. In addition, a few case reports describing isolated patients with FMS and CD simultaneously, all of whose symptoms dramatically resolved after the removal of gluten from their diet, have been reported [27,28].

Studies of the prevalence of CD in European patients with IBS are scant. Nevertheless, two separate prospective studies carried out in Sheffield, UK [29], and in Krakow, Poland [30], respectively, concluded that IBS patients were seven times more likely than matched controls to have biopsy-proven CD.

As previously stated, it is widely accepted that IBS and FMS are interrelated diseases, with IBS occurring in about 50% of FMS patients [4,7]. It is also well-known that the association of CD with IBS is not infrequent, because the prevalence of CD in IBS is four- to sevenfold higher than the prevalence of CD alone expected to be found in the general population [23,29,30].

CD is a multisystem autoimmune disorder related to a permanent intolerance of gluten stored in wheat, rye, barley and other cereals. It affects 1% to 2% of individuals (mainly females) worldwide. CD patients generally are carriers of one of the two major histocompatibility complex class II HLA genotypes, HLA-DQ2 or HLA-DQ8. In these patients, gliadin peptides trigger an aberrant immune response that results in the production of tTG autoantibodies and an immune-mediated chronic inflammation of the small-bowel mucosa. This immune-mediated enteropathy is characterized by villous atrophy, intraepithelial lymphocytosis and crypt hyperplasia. CD clinical manifestations may appear at any age together with gastrointestinal and/or extraintestinal systemic symptoms, although some diagnoses can be made in asymptomatic individuals. Notably, adhering to a GFD results in complete clinical remission and full intestinal mucosa recovery in the vast majority of CD patients [31,32].

The available level of scientific evidence supporting the association of CD with FMS is mostly limited to a low-powered, cross-sectional study in which a 2% incidence of CD was detected among 50 American adolescents with FMS [27], an American nationwide survey reported that FMS was the physician’s initial diagnosis in 9% of 134 patients later diagnosed with CD [25]. Two case reports in the literature have described, respectively, a child [27] and three women [28] with comorbid FMS and CD, all of whose symptoms resolved very well once gluten was removed from their diet. In the present study, we found that 6.7% patients with the triad of IBS, FMS and CD showed remarkable symptom improvement when placed on a GFD.

FMS is a complex chronic pain syndrome affecting 1% to 3% of people worldwide. FMS often clusters in families and affects mainly females (85% or more) between their 20s and 50s, although the incidence rises with age, reaching 7% in women over 70 years of age. Clinically, FMS is characterized by widespread soft-tissue pain; generalized TPs; abnormal fatigue; sleep disturbance; and skin, gastrointestinal, urinary, cognitive and various other symptoms. FMS symptoms vary from person to person in both number and severity and tend to fluctuate in response to emotional or physical stress, lack of sleep, exertion, injuries, infections, menstruation and weather changes, among other stressors. Because no effective treatment for FMS control is currently available, its personal, familial, labour-related and social impacts are very negative, usually leading to excessive use of healthcare services [6,33,34]. The pathogenesis of FMS remains elusive, although it is believed that genetic, immunologic and environmental factors contribute to its complex, multifactorial pathological process, and it is accepted that a combination of increased peripheral impulse input and increased central pain sensitivity, with aberrant pain facilitation and impaired inhibition, may be responsible for this disorder. The relative role of peripheral and central factors and ascending and descending pathways are not known, but probably all of the neural pathways, in variable proportions and depending of each case, may be involved in an interactive way [35,36]. No laboratory or imaging techniques for an accurate diagnosis of FM are currently available, thus necessitating an exclusion-based differential diagnosis to rule out other, similarly typified diseases after appropriate evaluation screening in patients fulfilling the 1990 ACR criteria for FMS [6]. Interestingly, FMS is frequently associated with other rheumatic disorders, infections and systemic illnesses. For example, 30% of females with ankylosing spondylitis, 16% with SLE, 15% with rheumatoid arthritis, 24% with psoriatic arthritis, 11% with osteoarthritis, 9.2% with Behçet’s disease and up to 40% with joint hypermobility syndrome meet the ACR criteria for FMS. FMS has also been documented in women with hyperprolactinaemia (71%), hypothyroidism (34%), Crohn’s disease (26%), ulcerative colitis (11%), diabetes mellitus (17%) and endometriosis (6%). An increased prevalence of FMS has also been reported in patients with hepatitis C virus (57%) and HIV (29%), as well as following physical trauma in 22% of patients, mostly women, with neck injuries and in 1.7% patients with lower-extremity fractures [33,34,36,37]. FMS is frequently found to be associated with other central sensitivity syndromes, such as: post-traumatic stress disorder (57%), CFS (55%), multiple chemical sensitivities (55%), IBS (41%), tension, migraine and mixed headaches (26%), TMJ disorder (24%), bulbar vestibulitis syndrome (23%), Gulf War syndrome (18%) and interstitial cystitis (15%) [33,36].

A separate issue is the importance of the significant number of IBS/FMS patients with related CD in our series who exhibited an excessive prevalence of ANA, anti-TPO and AMA autoantibodies. Indeed, there are two populations included in the present study: one constituted 104 IBS/FMS patients and the other 125 patients with only IBS. The IBS/FMS group had more frequent positive ANAs than those with only IBS (28% vs. 3%; *P* < 0.001), and the same was true for anti-TPO antibodies (11% vs. 0%), AMAs (5% vs. 0%) and anti-tTG autoantibodies (mean serum levels = 0.4 vs. 4.7; *P* < 0.001). However, none of the ANA-positive participants met the 1982 ACR criteria necessary for the diagnosis of SLE, none with increased anti-TPO antibodies showed clinical manifestations of thyroid dysfunction and none of the AMA-positive individuals had clinical manifestations of liver disease.

Because FMS cannot be considered an autoimmune disease, despite the fact that it is common in patients with autoimmune disease and may be the source of many of the symptoms and much of the disability in these patients [38], the findings of our study are more concordant with CD, an autoimmune disease often associated with a variety of autoimmune diseases, including type 1 diabetes mellitus, autoimmune thyroid disease, autoimmune adrenal disease, Sjögren syndrome, rheumatoid arthritis, SLE, primary biliary cirrhosis, primary sclerosing cholangitis, autoimmune cholangitis, autoimmune hepatitis and IBS [32].

A secondary finding worthy of comment is the large number of patients (20 in the IBS-only group and 58 in the IBS/FMS group) with increased infiltration of IELs (above 25% of enterocytes) with normal villous architecture in the duodenal mucosa, a pathologic finding known as *lymphocytic duodenosis* (LD). This condition, also named *lymphocytic duodenitis*, *nonatrophic lymphocytic enteritis* or (in the gluten-sensitivity context) *Marsh stage 1*, is an unspecific diagnosis of gluten intolerance by itself. Therefore, such patients should not be diagnosed with CD solely on the basis of histological studies. A recent prospectively designed study demonstrated that only 16% of patients with LD will develop CD and that the IEL count even becomes normal on repeated biopsies in up to 76% of patients [39]. In addition, different studies have reported a variety of LD and non-gluten-related sensitivity associations, such as drug intake (for example, nonsteroidal anti-inflammatory drugs (NSAIDs)), nongluten food protein intolerance (for example, cow’s milk, eggs, peanuts and soy), autoimmune disorders (for example, thyroiditis, type 1 diabetes mellitus, rheumatoid arthritis, psoriasis, multiple sclerosis and SLE), several inflammatory and/or infectious digestive disorders (for example, Crohn’s disease, *H. pylori*, bacterial overgrowth, tropical sprue, *Giardia lamblia*, *Cryptosporidium* and viral infections), IgA deficiency and T-cell intestinal lymphoma [40,41].

About 80% of the IBS/FMS patients in our series were taking NSAIDs on an irregular basis, and more than 40% from both cohorts were infected with *H. pylori* at the beginning of the study. Therefore, it appears reasonable to attribute the high prevalence of NSAID chronic intake and *H. pylori* infection found in our series to these two known confounder causes of LD. However, it should be taken into account that ten (17%) of these IBS-LD/FMS patients who tested positive for HLA-DQ2 A1/B1 and were also reported to have first-degree relatives with CD, which are two laboratory and clinical features compatible with a CD diagnosis.

The comorbid triad of IBS, chronic fatigue and musculoskeletal pain is striking [26], and the fact that our patients reported a long history of digestive complaints before the appearance of generalized soft-tissue pain, multiple TPs, weakness and other multisystemic symptoms resembling FMS is concordant with the increased prevalence of FMS reported by other series of women with different chronic processes within the gastrointestinal tract, such as IBS, Crohn’s disease and ulcerative colitis [33,36] and makes it feasible to hypothesize that a gluten-related autoimmune inflammatory process initiated within the gastrointestinal tract may contribute, in some gluten-sensitive patients with CD, to the well-documented central nervous system sensitivity (with aberrant pain facilitation and descending inhibitory pain impairment) responsible for FMS.

Despite the fact that our study included a relatively small number of nonrandomized patients comprising individuals referred to a gastroenterological clinic for symptoms indicative of a risk for CD, making it difficult to draw unequivocal conclusions, it appears evident that our findings may provide insightful contributions into the complex clinical picture of IBS/FMS CD-associated disorder, adding new clinical evidence in favour of the presence of a possible relationship between CD (and gluten sensitivity in general), IBS and some cases of FMS. Unfortunately, there are not any other available published case-finding studies of adult patients with FMS to establish comparisons.

## Conclusion

The findings of this case-finding study indicate that some misdiagnosed IBS/FMS patients could have underlying CD, which could contribute to IBS/FMS symptom development and maintenance. If they were replicated in the future with more potent studies, our observations could have pivotal therapeutic connotations, because one of the most disappointing aspects of FMS therapy is that only a small number of patients experience substantial relief when treated with the available US Food and Drug Administration–approved medications (that is, pregabalin, duloxetine and milnacipran), but a great number of patients discontinue their treatment because of drug-related adverse effects or its low effectiveness [42-45].

The detection of CD-IBS/FMS-like index cases implies that decisive therapeutic and preventative actions be taken for these patients and their relatives with silent or symptomatic, but not yet diagnosed, CD. These measures would include the implementation of a GFD, which can potentially improve symptoms, reverse the intestinal mucosal pathological changes and prevent long-term CD-related complications [31,32]. In addition, it is important to provide genetic counselling to patients and their families to help them understand the medical, psychological and familial implications of this disease; educate them about management, inheritance, prevention, testing and identification of reliable sources of information and products; and support adherence to a lifelong GFD regimen [46].

## Abbreviations

ACR: American College of Rheumatology; CD: Coeliac disease; CFS: Chronic fatigue syndrome; FIQ: Fibromyalgia Impact Questionnaire; FMS: Fibromyalgia syndrome; HAQ: Health Assessment Questionnaire; HLA: Human Leucocyte Antigen; IBS: Irritable bowel syndrome; iFOBT: Immunological faecal occult blood test; MHC: Major Histocompatibility Complex; SF-36: 36-Item Short Form Health Survey; TMJ: Temporomandibular joint; TP: Tender point.

## Competing interests

The authors declare that they have no competing interests.

## Authors’ contributions

LR designed the study and collected the patients and the clinical information. IB conceived the study, participated in its design and coordination and helped to draft the manuscript. JB performed all the clinical health-related QoL tests and calculated and interpreted the information derived from them. FdS participated in the design of the study, made interesting suggestions about the study design and contributed to the analysis and interpretation of the results. All authors read and approved the final manuscript.
